# First Report on the Occurrence and Subtypes of *Blastocystis* in Pigs in Poland Using Sequence-Tagged-Site PCR and Barcode Region Sequencing

**DOI:** 10.3390/pathogens9070595

**Published:** 2020-07-21

**Authors:** Monika Rudzińska, Beata Kowalewska, Beata Szostakowska, Maciej Grzybek, Katarzyna Sikorska, Agnieszka Świątalska

**Affiliations:** 1 Department of Tropical Medicine and Epidemiology, Institute of Maritime and Tropical Medicine, Medical University of Gdańsk, 81-519 Gdynia, Poland; bkowal@gumed.edu.pl (B.K.); ksikorska@gumed.edu.pl (K.S.); 2 Department of Tropical Parasitology, Institute of Maritime and Tropical Medicine, Medical University of Gdańsk, 81-519 Gdynia, Poland; bszost@gumed.edu.pl (B.S.); maciej.grzybek@gumed.edu.pl (M.G.); 3 Veterinary Hygiene Station, 80-316 Gdańsk, Poland; a.swiatalska@gdansk.wiw.gov.pl

**Keywords:** *Blastocystis*, pigs, Poland, molecular methods, epidemiology

## Abstract

*Blastocystis* is an enteric microorganism commonly found in humans and animals worldwide. Its pathogenic role in humans and transmission patterns has not been fully explained. However, nine subtypes (ST1–8, ST12) are considered as potentially zoonotic. Studies from various regions of the world show that pigs are mainly infected with ST5. Although pigs are important farmed animals in Poland, the question of *Blastocystis* infection in these animals has not yet been investigated. Herein, 149 pig stool samples from 10 Polish pig farms were analyzed using sequence-tagged-site PCR and barcode region sequencing. The percentage of samples in which *Blastocystis* was identified using each method separately was similar: 38.25% and 37.58%, respectively. However, the percentage of positive results obtained by combining both methods was 46.97%, which means that, depending on the method used, the number of undetected samples varied between 8.72% and 9.39%. This shows the methodological limitations of up-to-date molecular approaches commonly used in *Blastocystis* research. A moderate infection rate (44.4–50%) observed in different pig age groups with a vital predominance of ST5 (94.28%) in every age group shows that pigs are a likely natural host of ST5. A small percentage of mixed infections, namely ST5/ST1 (5.26%), ST5/ST3 (1.75%), and ST3/ST1 (1.75%), was observed only in animals of older age, suggesting that ST3 and ST1 can be acquired by pigs during contact with humans. This study provides the first data on the prevalence and *Blastocystis* subtypes (STs) distribution in pigs in Poland. The results also highlight the need for the development of new methods capable of detecting highly genetically diverse *Blastocystis* isolates and mixed infections.

## 1. Introduction

*Blastocystis* is a common enteric eukaryotic microorganism distributed worldwide, colonizing probably more than one billion people and a wide variety of mammals (wild, farm, and companion animals) as well as birds and insects [1,2,3]. *Blastocystis* pathogenicity and importance to public health is controversial since the organism is found in both asymptomatic and symptomatic individuals [4]. In the latter group, *Blastocystis* has been linked to gastrointestinal disorders (such as nausea, abdominal pain, diarrhoea, constipation, flatulence) as well a contribution to the development of irritable bowel syndrome (IBS) and irritation bowel disease (IBD), or rarely to skin reactions (urticaria, pruritus) [5,6,7]. *Blastocystis* exhibits extensive genetic diversity within the small subunit of ribosomal RNA (SSU rDNA) gene [8]. Studies on the sequence similarity within the SSU rDNA led to the identification of at least 17 microscopically indistinguishable subtypes named ST1–ST17 [9]. Nine of them (ST1–ST8, ST12) are found in both humans and animals [1,10], hence the suspicion that these subtypes may have zoonotic potential. However, neither the contribution of the animal reservoir to human infection nor the way of transmission has been fully clarified [11]. Contact with animals, their faeces, or water contaminated with cysts probably promote transmission, which presumably takes place via the faecal-oral route [12]. These pathways of infection have been confirmed by a higher frequency of *Blastocystis* infection in animal handlers [13,14] as well as by numerous reports showing that humans and animals remaining in close contact with each other were hosts of the same *Blastocystis* STs [15,16,17,18,19]. Identification of *Blastocystis* in pig manure slurry [20], sewage [21], and untreated drinking water [22,23] also indicate the possibility of being infected this way. Pigs, among many animal hosts, are often colonized by *Blastocystis* (from 7.5% to 100% in different world regions), and the most common subtype found in pigs is ST5 (for review see [24]). In some locations in Asia, this subtype (generally rare in humans) has been detected in people who had contact with pigs, suggesting a possible pattern of zoonosis [15,25,26,27]. In Poland, which is the fifth-largest producer of pork in Europe (with a pig population over 11 million heads) [28], *Blastocystis* is the most frequently detected eukaryotic organism in human stool samples [29]. Six *Blastocystis* subtypes (ST1–ST4, ST6, and ST7) have been reported in Poles by various authors [30,31,32,33] whereas large-scale animal testing has not been yet carried out.

*Blastocystis* is a highly polymorphic organism. At least 4 morphological forms differing in appearance and body size range were described [12,34]. Due to this, as well as a delicate, easily destructive structure, the detection of *Blastocystis* in routine microscopic stool smears is difficult and often unreliable since *Blastocystis* cells are easily overlooked or confused with other microorganisms present in faeces. The use of molecular methods significantly improved the sensitivity and specificity of *Blastocystis* detection in stool samples [14,17,35]. Of the several PCR-based methods, two techniques are most commonly used by *Blastocystis* researchers. The first involves the use of primers complementary to sequence-tagged-sites (STS), i.e., short, easy-to-recognize single DNA sequences specific for individual *Blastocystis* subtypes [36]. The vital drawback of the “STS method” is the necessity of performing separate PCR reactions with all available STS primer pairs for each sample and the ability to detect only seven *Blastocystis* subtypes, but its advantage is the ability for detecting mixed infections. The second is based on the amplification of a gene fragment encoding the 18S rRNA subunit (known as the barcode region), followed by sequencing of the resulting DNA amplification product [37]. In the “barcode method”, it is sufficient to perform only one amplification reaction for each sample, then the PCR product is sequenced and the subtype of *Blastocystis* can be further identified using programs available online (e.g., http://publmst.org/blastocystis/). This method allows detection of all the 17 so far identified *Blastocystis* subtypes as well as potentially novel ones, but it fails in case of mixed infections.

The study aimed to obtain the first data on the prevalence and subtypes of *Blastocystis* in pigs in Poland to better understand the host specificity and epidemiology of *Blastocystis* infection. In order not to miss any *Blastocystis* subtype (particularly in mixed infections), we used both the above-mentioned PCR methods in our study.

## 2. Results

**Figure 1:**
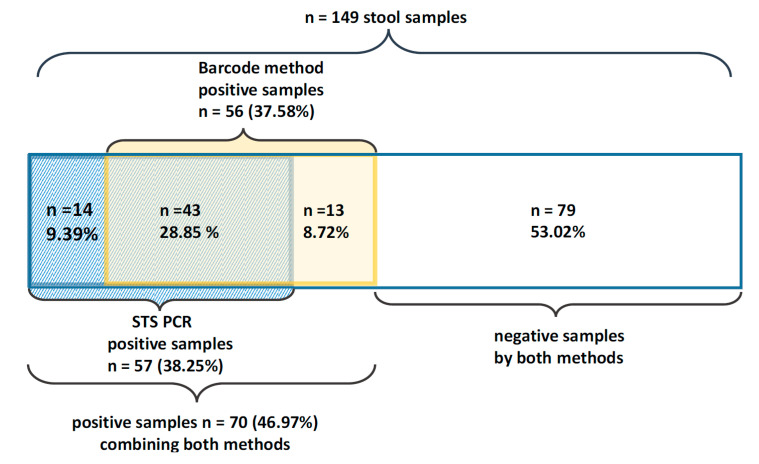
Comparison of *Blastocystis* detection results by STS PCR and barcode sequencing analysis.

After testing 149 pig stool samples for *Blastocystis* using two PCR research methods, the results obtained by each method were not completely consistent. *Blastocystis* DNA was detected in 57 samples (38.25%) tested with STS primers and 56 (37.58%) samples tested with barcode region analysis. In 43 samples (28.85%), *Blastocystis* was detected consistently with both methods used, whereas in 14 samples (9.39%), *Blastocystis* was found using only STS PCR, and in 13 (8.72%) using only “barcode method”. After adding up the results obtained with one or both of these methods, the actual number of *Blastocystis*-positive samples was 70 (46.97%). There were no statistical differences neither between the results obtained by two methods used in our study nor between each of these methods and the results obtained by the combination of both (*p* > 0.05). In 79 samples (53.02%), *Blastocystis* was not detected with any method (Figure 1). 

### 2.1. Results of STS PCR

Among all *Blastocystis* positive samples detected with STS PCR, 51 out of 57 samples (89.47%) represented ST5 and one in 57 (1.75%) with ST1. Five mixed infections were also detected: ST5/ST1 (three in 57, 5.26%), ST5/ST3 (one in 57, 1.75%), ST3/ST1 (one in 57, 1.75%). Summarizing the above, ST5 was found in 55 out of 57 (96.49%), ST1 in five out of 57 (8.77%), and ST3 in two out of 57 (3.51%) of samples (the sum of the percentages does not add up to 100% because of mixed infections) (Table 1).

### 2.2. Results of Barcode Region Analysis

Out of 66 samples that gave the product using barcode PCR, in 56 of them, obtained sequences corresponded to *Blastocystis*. Analysis of barcode region PCR products revealed ST5 in 52 out of 56 samples (92.85%) and ST1 in two of 56 samples (3.57%). The DNA sequences of these samples showed mostly high similarity (97–100%) to *Blastocystis* sequences deposited in GenBank. Sequences of the remaining two out of 56 samples (3.57%) (which gave no product with STS primers) had 87% and 76% similarity with *Blastocystis* sequences but not with a specific subtype. The sequence query facility recognized ST17 as the closest match to these sequences. However, due to low-quality of the DNA products the allocation remains highly uncertain. Hence, we classified these samples as *Blastocystis* ST indeterminate. In four cases the obtained sequences instead to *Blastocystis* corresponded to fungi of the genus *Galactomyces*, *Picchia*, and *Yarrowia*, while in six cases, the sequences were unreadable due to sequences overlapping and numerous gaps in the alignment. In all samples in which mixed infection was detected by STS primers, direct sequencing of the barcode region allowed to identify only one subtype: ST1 in a mixed sample of ST1/ST3, and ST5 in samples containing ST5/ST1 and ST5/ST3. In the case of 27 samples, the methods used gave inconsistent results. Details are presented in Table 1 and Figure 1.

In summary, analysis of the results of all 70 positive samples obtained with one of the two methods described above, or the combination of both, showed ST5 in 94.28% (66/70) of *Blastocystis* positive samples, ST1 in 7.14% (5/70), and ST3 in 2.85% (2/70). In two of 70 samples (2.85%), subtypes could not be identified.

### 2.3. Prevalence of Blastocystis and Subtypes Distribution within Pig Age Groups

**Figure 2:**
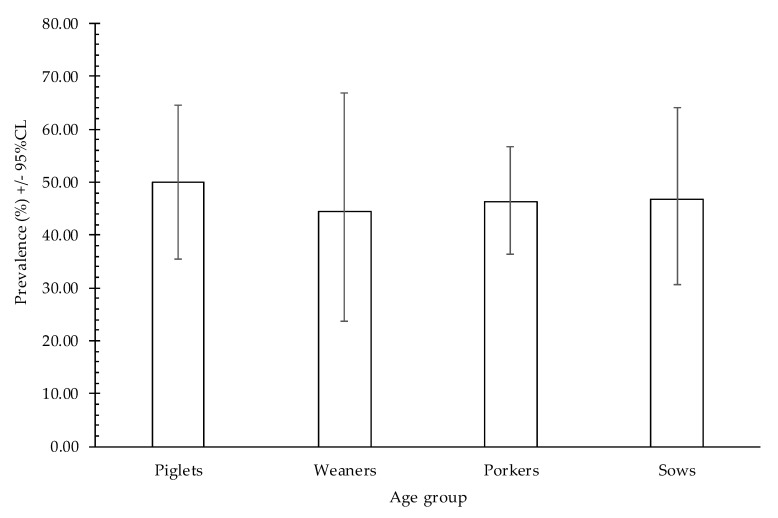
Prevalence of *Blastocystis* within pig age groups.

The overall prevalence of *Blastocystis* for all analyzed animals (n = 149) was 46.97% (95CL: 38.7–55.5%). *Blastocystis* colonized pigs from all age groups however, there was no significant difference in the prevalence between age groups (F_3,145_ = 0.57, *p* = 0.98). The percentage of infected animals was as follows: 50% in piglets aged <4 weeks, 44.4% in 1–3-month-old weaners, 46.3% in 3–9-month-old porkers, and 46.7% in >12-month-old sows (Figure 2).

ST5 was predominant in all age groups of pigs, with ST1 and mixed infections only found in isolated cases (Table 2).

## 3. Discussion

To the best of our knowledge, this is the first study providing information about *Blastocystis* prevalence and subtypes in pigs in Poland. *Blastocystis* has been reported as a common gut eukaryote in domestic pigs in several geographical regions of the world with the prevalence ranging from 7.5% to 100% [24]. 

In our study, the prevalence of *Blastocystis* in pigs was 46.97% which is similar to that obtained (also by PCR methods) in Spain (44.5%), Japan (44.1%), and Cambodia (45.2%), but lower than in most reports from Asian countries where infection rate often exceeds 70%, and in some of these countries it can even reach 100% [11,24,25,26,38,39]. These differences may be affected by the age of animals tested, the season of sampling for testing, the manner of animal keeping or environmental or sanitary conditions [25,39]. In comparison to abundant data from Asia on the prevalence of *Blastocystis* in pigs, data from European countries are scarce and highly diverse. In contrast to the mentioned study from Spain showing 44.5% of *Blastocystis* carriage in pigs, Alfelani et al. [11] reported 28.5% of infected pigs in the UK while Suli et al. [40] reported 81.2% in Serbia. These varied results do not allow a conclusion about the real *Blastocystis* prevalence in pigs in Europe and indicate the need for further research in this field.

Some authors noted the age of animals as a factor that could affect their susceptibility to *Blastocystis* infection. Pakandl et al. [41] noticed for the first time that colonization of *Blastocystis* had already begun in three-day-old piglets, reaching 90% in one-week-old piglets, and persisted at slightly varying levels (84–93%) for the rest of the animals’ life. The highest prevalence of *Blastocystis* in the youngest animals was observed by Navarro et al. [39] who explained this by the fact that very young animals without prior contact with *Blastocystis* are more susceptible to infection since they lack their sufficient immune response. On the contrary, the lowest prevalence observed in the youngest group of pigs was explained by effective immune protection due to the presence of maternal antibodies [42]. In our study, there was no significant difference between the age groups. The assessment of *Blastocystis* prevalence in pig age groups based on results obtained by different authors is not fully possible since age groups are defined differently in different studies.

To date, *Blastocystis* ST1–ST3 and ST5–ST7 have been reported in pigs worldwide with a definite predominance of ST5 followed by ST1 and ST3 [15,26,27,42,43,44]. ST10 was also reported, but only in one piglet in one study only [24]. Recently, ST15 was also identified in pig faecal samples [45]. In our study, ST5 was the most frequent subtype identified. However, apart from detecting ST5 and ST1, barcode region analysis also revealed two *Blastocystis*-positive samples which subtypes could not be determined probably because of the poor quality of the PCR products. It cannot be excluded that they contained *Blastocystis* subtypes other than ST1–ST7, which would explain the negative results obtained for these samples using STS primers. In addition to ST5 and ST1, ST3 was detected in two samples tested using STS primers. However, this subtype was present exclusively in a mixed infection together with ST5 or ST1. The ST3 was not detected in these samples using barcode method because direct sequencing of PCR product in mixed infections allows for detection of only the subtype whose DNA is predominant in the sample (here ST5 or ST1) [46]. For this reason, and because of the RD5 primer’s cross-reactivity with species other than *Blastocystis* [37], in three samples, sequencing revealed fungi of the genus *Galactomyces*, *Pichia*, and *Yarrowia* that are often present in the stool. Their detection, however, does not exclude the presence of *Blastocystis* in these samples, since ST5 was found in these samples using STS primers.

Despite our considerable effort, we failed to obtain PCR products in eleven samples using STS primers, although barcode region sequencing confirmed the presence of *Blastocystis* ST5 in these samples. Similar discrepancies about ST1–ST3 were observed by Stensvold [47]. STS primers were designed based on sequence data generated from randomly amplified polymorphic DNAs and still little is known about genetic diversity both between and within STS targets [46,48,49]. Therefore, it cannot be excluded that PCR products were not obtained because STS primers were not fully complementary to the binding sites in our DNA templates.

This plight has also occurred in the opposite direction, i.e., eight samples identified as *Blastocystis* ST5 with STS primers were negative with barcode PCR. Possibly, the worse quality of DNA templates of these samples caused that shorter DNA fragments (approximately ~300 bp) were successfully amplified (with STS primers) while amplification of the longer ~600 bp region failed, or it could be a sensitivity issue. Similar problems were encountered by Yoshikawa [50] when some samples positive with STS primers were negative in PCR with a 1.1 kbp SSU rDNA fragment.

Ultimately, the results of the study showed that, although the percentage of samples in which *Blastocystis* was identified using each method separately was almost identical, some samples determined as positive by one method remained undetected by the other one and vice versa. This shows that the use of anyone of these commonly adopted approaches separately may potentially lead to false-negative results in some samples. In our study, the underestimation of positive samples was 8.72% with STS PCR and 9.39% with barcode PCR and sequencing concerning the actual number of samples containing *Blastocystis* (Figure 1). However, the differences were not statistically significant (*p* > 0.05). This inconsistency in the results obtained using each method also highlighted what a challenge is to design *Blastocystis* specific primers [46,51].

In the recent decade, *Blastocystis* prevalence and subtypes in pigs have been studied mainly in countries of Southeast Asia [15,24,25,26,27,42,43] and a lesser extent in Australia [25,51] and USA [52,53]. To date, the most common subtype reported in pigs in these regions was ST5. In our study, ST5 was also the most frequently identified subtype, which suggests that there is no geographic restriction in the occurrence of ST5 in pigs. However, more research from Europe as well as other world regions are needed to confirm this observation. Scarce data from Europe showed the predominance of ST5 in pigs in Denmark [16] while ST1 was the most prevalent subtype in France [54] and Spain [39]. These data are difficult to assess and compare since they are mostly from before 2013 and apply to small groups of animals (≤20). The largest examined group (from Spain) consisted of 395 pigs. However, only eight of 122 *Blastocystis*-positive samples were randomly selected for sequencing [39]. In turn, authors from Serbia [40] who examined a group of 48 animals did not determine *Blastocystis* subtypes but focused on the frequency of *Blastocystis* in this group.

Apart of pigs, ST5 was also identified in other livestock animals including cattle [11,16,55,56], goats [57,58], sheep [44,59], and poultry [60]. However, in these groups of animals, ST5 was less common than in pigs. On the other hand, some authors did not found ST5 in these animals [11,43,44,61]. This suggests that pigs are the main host of ST5 and probable source of *Blastocystis* infection for other livestock animals which are often kept together or have contact with each other on farms. The sporadic detection of ST6 and ST7 in pigs (subtypes mostly found in birds, including domestic birds [16,62]) can denote that these subtypes, although specific for birds, could have been acquired by pigs through faecal-oral route [26]. This shows the probability of easy transmission of *Blastocystis* between different animal species.

Humans have been shown to harbour ten *Blastocystis* STs (ST1–ST9, ST12) but the majority of infections (~90%) are caused by four subtypes with a predominance of ST3 followed by ST1, ST2, and ST4, with proportions varying by geographical region. To date, ST5 has been sporadically reported in humans [63]. A handful of reports suggest that humans could have acquired this subtype by contact with pigs [15,25,27,38]. On the other hand, *Blastocystis* transmission in the opposite direction, i.e., from humans to pigs, seems to occur as well [26,27]. The following examples best describe different variants of this probably two-way transmission.

In a study in pigs and their in-contact humans, performed by Wang et al. [25], all *Blastocystis* positive pigs had ST5. Additionally, ST1 and ST3 were found in a small percentage of samples, and exclusively in mixed infection with ST5. Inversely, piggery workers harboured mainly ST3 and ST1, although mixed infection with ST5/ST3 or ST5 alone was found in some of them. Moreover, a comparison of the sequences of ST1, ST3, and ST5 obtained from pigs and their in-contact humans revealed 100% identity in the case of ST1 and ST3, as well as almost 100% (with a single nucleotide polymorphism) in the case of ST5, suggesting possible cross-infection with these subtypes between pigs and their caregivers.

Similarly, Pintong et al. [27] noticed that some sequences of ST5 and ST1 found in pigs and people working on or living near pig farms were highly similar (98–99%) to each other as well as to sequences of ST5 and ST1 derived from humans, pigs and cattle, previously reported in GenBank, which suggest the possibility of multi-directional transmission of these subtypes between livestock animals and people.

In another study, apart from ST5 that was found in all pigs in the study group, the authors also found ST1 in 16.4% and ST2 in 11% of animals. These two subtypes were common in children living in an area where the study was conducted. Interestingly, none of the children harboured ST5 despite the poor hygiene conditions prevailing in this village [26]. However, Udonsom et al. [43] reported that people living around the pig farms did not host ST5, which was found in pigs, and vice-versa–ST1, ST2, or ST3, although present in these people, were not detected in pigs. When compared to the GenBank database, only one ST5 isolated from a pig was closely related to *Blastocystis* sequence derived from a human, which shows that transmission between humans and animals does not always occur.

These examples show that, although pigs seem to be a natural host for ST5, this subtype can adapt to a human host, and can be transmitted from pigs to humans, which may be favoured by poor sanitation, improper waste management in pig farms, lack of personal hygiene, or no access to clean potable water [25,27]. Although, as shown above, contact or sharing living space with pigs does not always result in such a transmission.

On the other hand, the presence of ST3 (which predominates in humans) and ST1 (also common in humans) in pigs indefinitely smaller quantities than ST5, and often in mixed infection together with ST5 (as took place in our own and some of the mentioned studies), suggests that pigs probably acquire these subtypes by contact with humans. The ability of *Blastocystis* to be transmitted from humans to pigs was noticed for the first time in 1993 by Pakandl et al. [64] who performed the successful experimental transfer of a human *Blastocystis* isolate (of unknown ST) to a gnotobiotic piglet (although only one of 16 piglets became infected).

An interesting observation in our study is that ST5 was the only subtype found in the youngest group of pigs (piglets up to four weeks old). In older groups, in addition to the presence of ST5, other *Blastocystis* subtypes were also detected either in mixed or homogenous infections. This suggests that older animals, i.e., those that have had longer contact with people, have acquired subtypes usually harboured by humans (Table 2).

To date, there is no Polish publication about the occurrence of *Blastocystis* in animals. Only a few conference reports are available regarding small groups of birds [65,66] and animals from the Wrocław Zoo [67]. Among them, ST5 was found in chickens and among zoo animals but the authors did not provide any information in what animal species. Livestock has not been examined so far. When it comes to humans, ST5 was not detected in any of the human samples tested in a few regions of Poland. However, no information is available on whether these people have had any contact with animals [30,31,32,33,68]. 

## 4. Materials and Methods

### 4.1. Stool Sampling and Processing

Between November 2017 and February 2018, 149 pig stool samples including 32 piglets (<4 weeks old), 18 weaners (1–3 months old), 54 porkers (3–9 months old) and 45 sows (>12 months old) were collected from 15 piggeries in the Pomerania Province. Then, ten samples were taken from each of 14 piggeries and nine samples from one piggery. One stool sample was taken from each animal pen. 

Stool samples were collected from the ground after animal defecation (from the top of droppings to minimize contamination), placed into plastic stool collection containers, transported to the Department of Tropical Medicine and Epidemiology (Medical University of Gdańsk) under cool conditions, then suspended in 70% ethanol and stored at 4 °C until DNA extraction. 

### 4.2. DNA Extraction and PCR Amplification 

Immediately before DNA extraction, alcohol was washed off the stool samples with phosphate-buffered saline (PBS) two times and then one time with sterile distilled water by centrifugation at 2000× *g* for 10 min. Each time the supernatant was discarded carefully with a pipette.

Genomic DNA was extracted from washed samples using Genomic Mini AX Stool Kit (A&A Biotechnology, Gdynia, Poland) according to the manufacturer’s instruction. All PCR templates were additionally treated with an Anti-Inhibitor Kit (A&A Biotechnology, Gdynia, Poland) to remove PCR inhibitors that could disturb reaction. Extracted DNAs were stored at −20 °C before PCR amplification.

For specific detection of *Blastocystis* two conventional PCR methods were performed:

(i) PCR with subtype-specific sequence-tagged-site (STS) diagnostic primers described by Yoshikawa et al. [36] (Table 3). DNA amplification was carried out in 25 μL reaction mixtures consisting of 12.5 μL PCR Mix HGC Plus (ready-to-use PCR mixture containing Taq DNA polymerase, PCR buffer, MgCl2, and dNTPs; A&A Biotechnology), 1 μL of each primer (concentration 10 μM), 2 μL of genomic DNA, supplemented with deionized water up to 25 μL. The PCR conditions were the same for all primer sets and consisted of the following steps: 3 min at 94 °C (initial denaturation) followed by 35 cycles of 30 s at 94 °C, 30 s at 59 °C, 1 min at 72 °C, and a final extension step of 5 min at 72 °C. STS PCR results were considered positive if a specific band was visible in the gel.
(ii) Amplification and sequencing of a ~600 bp fragment of the SSU rDNA gene (called barcode region) with the *Blastocystis*-specific BhRDr and the broad-eukaryote-specific RD5 primers described by Scicluna et al. [37] (Table 3). The reaction mixture was as above. The PCR conditions were as follows: 4 min at 94 °C (initial denaturation) followed by 35 cycles of 15 s at 95 °C, 15 s at 60 °C, 30 s at 72 °C, and a final extension step of 5 min at 72 °C.

Negative (water instead of extracted DNA) and positive (a sample successfully sequenced for *Blastocystis* before this study) control was placed in each amplification batch. PCRs were conducted in a thermal cycler (Mastercycler, Eppendorf, Hamburg, Germany). The PCR products were electrophoresed on a 1.5% agarose gel (Sigma, St. Louis, MO, USA) at 150 V for 40 min with Tris-borate-EDTA buffer. Bands were visualized by Midori Green DNA Stain (Nippon Genetics Europe, Duren, Germany) using an ultraviolet gel documentation system Gel Doc-It (UVP LLC, Upland, CA, USA).

### 4.3. Barcode Sequence Analysis

Direct sequencing of barcode region PCR products was performed in both directions by standard procedure with the primers used for amplification. The obtained sequences were then analyzed using GeneStudio Pro Software (GeneStudio Inc., Suwanee, GA, USA) and compared with sequences available in GenBank using NCBI BLAST (http://www.ncbi.nlm.nih.gov/BLAST). Barcode PCR results were considered positive if direct sequencing of the products confirmed *Blastocystis*. The chosen *Blastocystis* sequences identified in this study have been deposited in the GenBank database under accession numbers of MT373829 to MT373878. *Blastocystis* subtypes affiliation was determined using the sequence query facility at www.pubmlst.org/blastocystis recommended by Stensvold [47], which is under the consensus terminology for *Blastocystis* subtypes established in 2007 [69].

### 4.4. Statistical Analysis

Prevalence values (percentage of positive samples) are given with 95% confidence limits in parenthesis (CL95) or error bars on figures, calculated using bespoke software “PERCENTAGE CONFIDENCE LIMITS VS 13” (courtesy of Prof. F.S. Gilbert and Prof. J.M. Behnke, University of Nottingham) based on the tables of Rolf and Sokal [70,71]. Differences between age groups were analyzed using ANOVA. Differences between diagnostic methods were analyzed using Student’s *t*-test. Descriptive analysis (percentages) was used to recognize *Blastocystis* STs distribution.

## 5. Conclusions

To the best of our knowledge, this study is the first to evaluate the colonization and *Blastocystis* subtypes’ distribution in pigs in Poland. A moderate *Blastocystis* infection rate (44.4–50%) was observed in different age groups of pigs with a vital predominance of ST5 in every age group, suggesting that pigs are natural host of ST5. The observation that, in animals, mixed infections (ST5/ST1, ST3/ST1) appear with age may indicate that ST3 and ST1 could have been acquired by pigs during contact with humans. This shows clearly the need for further research in pigs and their in-contact humans from multiple world regions to understand the specificity and relationship of different *Blastocystis* STs within and between individual host groups. This will help to establish whether pigs are a reservoir of *Blastocystis* and determine their role in spreading this infection to humans.

The results also highlighted methodological limitations of up-to-date molecular approaches used commonly in *Blastocystis* identification, i.e., the possibility of missing some infections during diagnostics. Consequently, it also demonstrated the need to include in the assessment molecular methods less frequently used by researchers to identify *Blastocystis* in order to select and use a PCR method capable of detecting the highly genetically diverse *Blastocystis* isolates as well as mixed infections at one go.

## Figures and Tables

**Table 1:** Results obtained with STS PCR and barcode sequence analysis.

No of Samples n = 149	Barcode Sequence Analysis		STS PCR (Positive n = 57)
	Product of Barcode PCR (Positive n = 66)	Identification Based on Nucleotide Sequence (Positive n = 56 **)	
37	+	ST5	ST5
1	+	ST1	ST1
3	+	ST5	ST5/ST1
1	+	ST5	ST5/ST3
1	+	ST1	ST3/ST1
11	+	ST5	-
2	+	ST-In ***	-
3	+	fungus	ST5
3	+	unreadable	ST5
8	-	-	ST5
1 *	+	fungus	-
3 *	+	unreadable	-
75	-	-	-

* were included in *Blastocystis* negative giving the total number of 79 negative samples. ** nucleotide sequence corresponding to *Blastocystis*. *** subtype indeterminate.

**Table 2:** *Blastocystis* subtypes distribution within pig age groups.

Age Group	No. of Examined/Infected Pigs	*Blastocystis* ST (n)
Piglets <4 weeks	32/16	ST5 (16)
Weaners 1–3 months	18/8	ST5 (7), ST5/ST3 (1)
Porkers 3–9 months	54/25	ST5 (21), ST1 (1), ST5/ST1 (2), ST-In * (1)
Sows >12 months	45/21	ST5 (18), ST5/ST1 (1), ST3/ST1 (1), ST-In * (1)

* subtype indeterminate.

**Table 3:** Primers used in this study.

Target *Blastocystis* ST	Primer Sets	Sequences of Forward (F) and Reverse (R) Primers (5′ to 3′)
ST1 ^a^	SB83	F: GAAGGACTCTCTGACGATGA R: GTCCAAATGAAAGGCAGC
ST2 ^a^	SB340	F: TGTTCTTGTGTCTTCTCAGCTC R:TTCTTTCACACTCCCGTCAAT
ST3 ^a^	SB 227	F: TAGGATTTGGTGTTTGGAGA R: TTAGAAGTGAAGGAGATGGAAG
ST4 ^a^	SB 337	F: GTCTTTCCCTGTCTATTCTTGCA R: AATTCGGTCTGCTTCTTCTG
ST5 ^a^	SB336	F: GTGGGTAGAGGAAGGAAAACA R: AGAACAAGTCGATGAAGTGAGAT
ST6 ^a^	SB 332	F: GCATCCAGACTACTATCAACATT R: CCATTTTCAGACAACCACTTA
ST7 ^a^	SB 155	F: ATCAGCCTACAATCTCCTC R: ATCGCCACTTCTCCAAT
All	RD5	ATCTGGTTGATCCTGCCAGT
	BhRDr	GAGCTTTTTAACTGCAACAACG

^a^ according to consensus nomenclature by Stensvold et al. [69] which is different from that in the original primer description by Yoshikawa et al. [36].

## References

[1] Stensvold C.R., Clark C.G. (2016). Current status of Blastocystis: A personal view. Parasitol. Int..

[2] Yoshikawa H., Koyama Y., Tsuchiya E., Takami K. (2016). Blastocystis phylogeny among various isolates from humans to insects. Parasitol. Int..

[3] Jiménez P.A., Jaimes J.E., Ramírez J.D. (2019). A summary of *Blastocystis* subtypes in North and South America. Parasit. Vectors..

[4] Andersen L.O., Stensvold C.R. (2016). Blastocystis in Health and Disease: Are We Moving from a Clinical to a Public Health Perspective?. J. Clin. Microbiol..

[5] Poirier P., Wawrzyniak I., Vivarès C.P., Delbac F., El Alaoui H. (2012). New insights into *Blastocystis* spp.: A potential link with irritable bowel syndrome. PLoS Pathog..

[6] Bahrami F., Babaei E., Badirzadeh A., Riabi T.R., Abdoli A. (2019). Blastocystis, urticaria, and skin disorders: Review of the current evidences. Eur. J. Clin. Microbiol. Infect. Dis..

[7] Shirvani G., Fasihi-Harandi M., Raiesi O., Bazargan N., Zahedi M.J., Sharifi I., Kalantari-Khandani B., Nooshadokht M., Shabandoust H., Mohammadi M.A. (2019). Prevalence and Molecular Subtyping of Blastocystis from Patients with Irritable Bowel Syndrome, Inflammatory Bowel Disease and Chronic Urticaria in Iran. Acta Parasitol..

[8] Gentekaki E., Curtis B.A., Stairs C.W., Klimeš V., Eliáš M., Salas-Leiva D.E., Herman E.K., Eme L., Arias M.C., Henrissat B. (2017). Extreme Genome Diversity in the Hyper-Prevalent Parasitic Eukaryote Blastocystis. PLoS Biol..

[9] Stensvold C.R., Clark C.G. (2020). Pre-empting Pandora’s Box: *Blastocystis* Subtypes Revisited. Trends Parasitol..

[10] Wawrzyniak I., Poirier P., Texier C., Delbac F., Viscogliosi E., Dionigia M., Alaoui H.E. (2013). Blastocystis, an unrecognized parasite: An overview of pathogenesis and diagnosis. Adv. Infect. Dis..

[11] Alfellani M.A., Taner-Mulla D., Jacob A.S., Imeede C.A., Yoshikawa H., Stensvold C.R., Clark C.G. (2013). Genetic Diversity of Blastocystis in Livestock and Zoo Animals. Protist.

[12] Clark C.G., van der Giezen M., Alfellani M.A., Stensvold C.R. (2013). Recent Developments in Blastocystis Research.

[13] Salim H.R., Kumar G.S., Vellayan S., Mak J.W., Khairul Anuar A., Init I., Vennila G.D., Saminathan R., Ramakrishnan K. (1999). Blastocystis in animal handlers. Parasitol. Res..

[14] Dagci H., Kurt Ö., Demirel M., Mandiracioglu A., Aydemir S., Saz U., Bart A., Van Gool T. (2014). Epidemiological and diagnostic features of blastocystis infection in symptomatic patients in izmir province, Turkey. Iran. J. Parasitol..

[15] Yan Y., Su S., Ye J., Lai X., Lai R., Liao H., Chen G., Zhang R., Hou Z., Luo X. (2007). *Blastocystis* sp. subtype 5: A possibly zoonotic genotype. Parasitol. Res..

[16] Stensvold C.R., Alfellani M.A., Nørskov-Lauritsen S., Prip K., Victory E.L., Maddox C., Nielsen H.V., Clark C.G. (2009). Subtype distribution of Blastocystis isolates from synanthropic and zoo animals and identification of a new subtype. Int. J. Parasitol..

[17] Parkar U., Traub R.J., Vitali S., Elliot A., Levecke B., Robertson I., Geurden T., Steele J., Drake B., Thompson R.C.A. (2010). Molecular characterization of Blastocystis isolates from zoo animals and their animal-keepers. Vet. Parasitol..

[18] Nagel R., Cuttell L., Stensvold C.R., Mills P.C., Bielefeldt-Ohmann H., Traub R.J. (2012). *Blastocystis* subtypes in symptomatic and asymptomatic family members and pets and response to therapy. Intern. Med. J..

[19] Lee L.I., Chye T.T., Karmacharya B.M., Govind S.K. (2012). *Blastocystis* sp.: Waterborne zoonotic organism, a possibility?. Parasites Vectors.

[20] Snell-Castro R., Godon J.J., Delgenès J.P., Dabert P. (2005). Characterisation of the microbial diversity in a pig manure storage pit using small subunit rDNA sequence analysis. Fems Microbiol. Ecol..

[21] Suresh K., Smith H.V., Tan T.C. (2005). Viable Blastocystis cysts in Scottish and Malaysian sewage samples. Appl. Environ. Microbiol..

[22] Leelayoova S., Siripattanapipong S., Thathaisong U., Naaglor T., Taamasri P., Piyaraj P., Mungthin M. (2008). Drinking water: A possible source of *Blastocystis* spp. subtype 1 infection in schoolchildren of a rural community in central Thailand. Am. J. Trop. Med. Hyg..

[23] Noradilah S.A., Lee I.L., Anuar T.S., Salleh F.M., Manap S.N.A.A., Mohtar N.S.H.M., Azrul S.M., Abdullah W.O., Moktar N. (2016). Occurrence of *Blastocystis* sp. in water catchments at Malay villages and Aboriginal settlement during wet and dry seasons in Peninsular Malaysia. PeerJ.

[24] Song J.K., Hu R.S., Fan X.C., Wang S.S., Zhang H.J., Zhao G.H. (2017). Molecular characterization of Blastocystis from pigs in Shaanxi province of China. Acta Trop..

[25] Wang W., Owen H., Traub R.J., Cuttell L., Inpankaew T., Bielefeldt-Ohmann H. (2014). Molecular epidemiology of Blastocystis in pigs and their in-contact humans in Southeast Queensland, Australia, and Cambodia. Vet. Parasitol..

[26] Yoshikawa H., Tokoro M., Nagamoto T., Arayama S., Asih P.B.S., Rozi I.E., Syafruddin D. (2016). Molecular survey of *Blastocystis* sp. from humans and associated animals in an Indonesian community with poor hygiene. Parasitol. Int..

[27] Pintong A., Sunyanusin S., Prasertbun R., Mahittikorn A., Mori H., Changbunjong T., Komalamisra C., Sukthana Y., Popruk S. (2018). *Blastocystis* subtype 5: Predominant subtype on pig farms, Thailand. Parasitol. Int..

[28] GUS Pogłowie świń 2019. https://stat.gov.pl/obszary-tematyczne/rolnictwo-lesnictwo/produkcja-zwierzeca-zwierzeta-gospodarskie/poglowie-swin-wedlug-stanu-w-grudniu-2019-roku,7,12.html.

[29] Kowalewska B., Rudzińska M., Zarudzka D., Kotłowski A. (2013). Ocena częstości zarażeń pasożytami jelitowymi wśród pacjentów przychodni Instytutu Medycyny Morskiej i Tropikalnej w Gdyni w okresie ostatnich 30 lat An evaluation of the intensity of intestinal parasitic infections among patients of out-patient Division. Diagn. Lab..

[30] Wesolowska W., Kicia M., Szetela B., Kopacz Z., Salamatin R., Rymer W., Szymczak A., Knysz B. (2016). Prevalence of *Blastocystis hominis* among HIV-positive and HIV-negative patients in Poland. Prevalence.

[31] Lepczyńska M., Białkowska J., Dzika E., Piskorz-Ogórek K., Korycińska J. (2017). Blastocystis: How do specific diets and human gut microbiota affect its development and pathogenicity?. Eur. J. Clin. Microbiol. Infect. Dis..

[32] Kaczmarek A., Gołąb E., Żarnowska-Prymek H., Rawska A., Jańczak D., Lewicki A., Wesołowska M., Rożej-Bielicka W., Cielecka D., Sałamatin R. (2017). Genetic diversity of *Blastocystis hominis* sensu lato isolated from humans in Poland = Zróżnicowanie genetyczne *Blastocystis hominis* sensu lato wyizolowanych od ludzi w Polsce. Przegl. Epidemiol..

[33] Rudzińska M., Kowalewska B., Wąż P., Sikorska K., Szostakowska B. (2019). *Blastocystis* subtypes isolated from travelers and non-travelers from the north of Poland—A single center study. Infect. Genet. Evol..

[34] Tan K.S.W. (2008). New Insights on Classification, Identification, and Clinical Relevance of *Blastocystis* spp.. Clin. Microbiol. Rev..

[35] Dogruman-Al F., Simsek Z., Boorom K., Ekici E., Sahin M., Tuncer C., Kustimur S., Altinbas A. (2010). Comparison of methods for detection of Blastocystis infection in routinely submitted stool samples, and also in IBS/IBD Patients in Ankara, Turkey. PLoS ONE.

[36] Yoshikawa H., Wu Z., Kimata I., Iseki M., Ali I.K.M.D., Hossain M.B., Zaman V., Haque R., Takahashi Y. (2004). Polymerase chain reaction-based genotype classification among human *Blastocystis hominis* populations isolated from different countries. Parasitol. Res..

[37] Scicluna S.M., Tawari B., Clark C.G. (2006). DNA barcoding of Blastocystis. Protist.

[38] Thathaisong U., Worapong J., Tan-ariya P., Viputtigul K., Mungthin M., Sudatis A., Noonai A., Leelayoova S. (2003). Blastocystis Isolates from a Pig and a Horse Are Closely Related to *Blastocystis hominis* Blastocystis Isolates from a Pig and a Horse Are Closely Related to *Blastocystis hominis*. J. Clin. Microbiol..

[39] Navarro C., Domínguez-Márquez M.V., Garijo-Toledo M.M., Vega-García S., Fernández-Barredo S., Pérez-Gracia M.T., García A., Borrás R., Gómez-Muñoz M.T. (2008). High prevalence of Blastocystis sp. in pigs reared under intensive growing systems: Frequency of ribotypes and associated risk factors. Vet. Parasitol..

[40] Süli T., Kozoderović G., Potkonjak A., Simin S., Simin V., Lalošević V. (2018). Comparison of conventional and molecular diagnostic techniques for detection of *Blastocystis* sp. In pig faeces. Iran. J. Parasitol..

[41] Pakandl M. (1991). Occurrence of *Blastocystis* sp. in pigs. Folia Parasitol. (Praha)..

[42] Paik S., Jung B.Y., Lee H., Hwang M.H., Han J.E., Rhee M.H., Kim T.H., Kwon O.D., Kwak D. (2019). Molecular Detection and Subtyping of Blastocystis in Korean Pigs. Korean J. Parasitol..

[43] Udonsom R., Prasertbun R., Mahittikorn A., Mori H., Changbunjong T., Komalamisra C., Pintong A., Sukthana Y., Popruk S. (2018). Blastocystis infection and subtype distribution in humans, cattle, goats, and pigs in central and western Thailand. Infect. Genet. Evol..

[44] Wang J., Gong B., Yang F., Zhang W., Zheng Y., Liu A. (2018). Subtype distribution and genetic characterizations of Blastocystis in pigs, cattle, sheep and goats in northeastern China’s Heilongjiang Province. Infect. Genet. Evol..

[45] Wylezich C., Belka A., Hanke D., Beer M., Blome S., Höper D. (2019). Metagenomics for broad and improved parasite detection: A proof-of concept study using swine faecal samples. Int. J. Parasitol..

[46] Scanlan P.D., Stensvold C.R., Cotter P.D. (2015). Development and application of a *Blastocystis* subtype-specific PCR assay reveals that mixed-subtype infections are common in a healthy human population. Appl. Environ. Microbiol..

[47] Stensvold C.R. (2013). Comparison of sequencing (Barcode Region) and sequence-tagged-site PCR for *Blastocystis* subtyping. J. Clin. Microbiol..

[48] Yoshikawa H., Abe N., Wu Z. (2004). PCR-based identification of zoonotic isolates of Blastocystis from mammals and birds. Microbiology.

[49] Stensvold C.R., Alfellani M., Clark C.G. (2012). Levels of genetic diversity vary dramatically between *Blastocystis* subtypes. Infect. Genet. Evol. J. Mol. Epidemiol. Evol. Genet. Infect. Dis..

[50] Yoshikawa H., Dogruman-AI F., Turk S., Kustimur S., Balaban N., Sultan N. (2011). Evaluation of DNA extraction kits for molecular diagnosis of human *Blastocystis* subtypes from fecal samples. Parasitol. Res..

[51] Roberts T., Stark D., Harkness J., Ellis J. (2013). Subtype distribution of Blastocystis isolates from a variety of animals from New South Wales, Australia. Vet. Parasitol..

[52] Santín M., Gómez-Muñoz M.T., Solano-Aguilar G., Fayer R. (2011). Development of a new PCR protocol to detect and subtype *Blastocystis* spp. from humans and animals. Parasitol. Res..

[53] Fayer R., Elsasser T., Gould R., Solano G., Urban J., Santin M. (2014). Blastocystis tropism in the pig intestine. Parasitol. Res..

[54] Noël C., Peyronnet C., Gerbod D., Edgcomb V.P., Delgado-Viscogliosi P., Sogin M.L., Capron M., Viscogliosi E., Zenner L. (2003). Phylogenetic analysis of *Blastocystis* isolates from different hosts based on the comparison of small-subunit rRNA gene sequences. Mol Biochem Parasitol..

[55] Badparva E., Sadraee J., Kheirandish F. (2015). Genetic Diversity of Blastocystis Isolated from Cattle in Khorramabad, Iran. Jundishapur J. Microbiol..

[56] Weining Z., Tao W., Gong B., Yang H., Li Y., Song M., Lu Y., Li W. (2017). First report of Blastocystis infections in cattle in China. Vet. Parasitol..

[57] Song J.K., Yin Y.L., Yuan Y.J., Tang H., Ren G.J., Zhang H.J., Li Z.X., Zhang Y.M., Zhao G.H. (2017). First genotyping of *Blastocystis* sp. in dairy, meat, and cashmere goats in northwestern China. Acta Trop..

[58] Betts E.L., Gentekaki E., Thomasz A., Breakell V., Carpenter A.I., Tsaousis A.D. (2017). Genetic diversity of Blastocystis in non-primate animals. Parasitology.

[59] Li W.C., Wang K., Gu Y. (2018). Occurrence of *Blastocystis* sp. and *Pentatrichomonas hominis* in sheep and goats in China. Parasit. Vectors.

[60] Valença-Barbosa C., Do Bomfim T.C.B., Teixeira B.R., Gentile R., Da Costa Neto S.F., Magalhães B.S.N., De Almeida Balthazar D., Da Silva F.A., Biot R., D’Avila Levy C.M. (2019). Molecular epidemiology of Blastocystis isolated from animals in the state of Rio de Janeiro, Brazil. PLoS ONE.

[61] Tan T.C., Tan P., Sharma R., Sugnaseelan S., Suresh K. (2013). Genetic diversity of caprine Blastocystis from Peninsular Malaysia. Parasitol. Res..

[62] Greige S., El Safadi D., Bécu N., Gantois N., Pereira B., Chabé M., Benamrouz-Vanneste S., Certad G., El Hage R., Chemaly M. (2018). Prevalence and subtype distribution of *Blastocystis* sp. isolates from poultry in Lebanon and evidence of zoonotic potential. Parasites Vectors.

[63] Alfellani M.A., Stensvold C.R., Vidal-Lapiedra A., Onuoha E.S.U., Fagbenro-Beyioku A.F., Clark C.G. (2013). Variable geographic distribution of *Blastocystis* subtypes and its potential implications. Acta Trop..

[64] Pakandl M., Koudela B., Vitovec J. (1993). An experimental infection of conventional and gnotobiotic piglets with human and porcine strains of Blastocystis. Folia Parasitol. (Praha).

[65] Lewicki A., Rożej-Bielicka W., Sałamatin R. (2016). *Blastocystis hominis* s. l. ST6—Parasite of chickens—New zoonotic agent in Poland. Ann. Parasitol..

[66] Kaczmarek A., Lewicki A., Dziedzic K., Sulecki K., Rożej-bielicka W., Wesołowska M., Gołąb E. (2019). A survey of Blastocystis in domestic chickens from Poland and Madagascar. Ann. Parasitol..

[67] Wesołowska M., Paszta W., Michrowska A., Piekarska J., Wesołowska M., Gorczykowski M., Kaczmarek A., Sałamatin R. (2019). Molecular characterization of *Blastocystis* subtypes isolated from various mammalian groups living in Wrocław ZOO, Poland. Ann. Parasitol..

[68] Sałamatin R., Kaczmarek A., Rożej-bielicka W., Cielecka D., Jańczak D., Lewicki A., Wesołowska M., Młocicki D., Gołąb E. (2016). Genotype characterisation of Blastocystis isolates from Polish patients—Preliminary results. Ann. Parasitol..

[69] Stensvold C.R., Suresh G.K., Tan K.S.W., Thompson R.C.A., Traub R.J., Viscogliosi E., Yoshikawa H., Clark C.G. (2007). Terminology for *Blastocystis* subtypes—A consensus. Trends Parasitol..

[70] Rolf F.J., Sokal R.R. (1995). Statistical Tables.

[71] Grzybek M., Bajer A., Bednarska M., Al-Sarraf M., Behnke-Borowczyk J., Harris P.D., Price S.J., Brown G.S., Osborne S.-J., Siński E. (2015). Long-term spatiotemporal stability and dynamic changes in helminth infracommunities of bank voles (*Myodes glareolus*) in NE Poland. Parasitology.

